# Diving deep into trouble: the role of foraging strategy and morphology in adapting to a changing environment

**DOI:** 10.1093/conphys/coaa111

**Published:** 2020-12-28

**Authors:** Monique Ladds, David Rosen, Carling Gerlinsky, David Slip, Robert Harcourt

**Affiliations:** 1 Marine Ecosystems Team, Department of Conservation, Wellington 6011, New Zealand; 2 Marine Predator Research Group, Department of Biological Sciences, Macquarie University, North Ryde 2113, Australia; 3 Marine Mammal Research Unit, Institute for the Oceans and Fisheries, University of British Columbia, Vancouver V6T 1Z4, Canada; 4 Taronga Conservation Society Australia, Mosman 2088, Australia

**Keywords:** Otariid, aerobic dive limit, prey availability, meta-analysis, diving

## Abstract

Physiology places constraints on an animal’s ability to forage and those unable to adapt to changing conditions may face increased challenges to reproduce and survive. As the global marine environment continues to change, small, air-breathing, endothermic marine predators such as otariids (fur seals and sea lions) and particularly females, who are constrained by central place foraging during breeding, may experience increased difficulties in successfully obtaining adequate food resources. We explored whether physiological limits of female otariids may be innately related to body morphology (fur seals vs sea lions) and/or dictate foraging strategies (epipelagic vs mesopelagic or benthic). We conducted a systematic review of the increased body of literature since the original reviews of Costa *et al.* (When does physiology limit the foraging behaviour of freely diving mammals? *Int Congr Ser* 2004;**1275**:359–366) and Arnould and Costa (Sea lions in drag, fur seals incognito: insights from the otariid deviants. In *Sea Lions of the World Fairbanks*. Alaska Sea Grant College Program, Alaska, USA, pp. 309–324, 2006) on behavioural (dive duration and depth) and physiological (total body oxygen stores and diving metabolic rates) parameters. We estimated calculated aerobic dive limit (cADL—estimated duration of aerobic dives) for species and used simulations to predict the proportion of dives that exceeded the cADL. We tested whether body morphology or foraging strategy was the primary predictor of these behavioural and physiological characteristics. We found that the foraging strategy compared to morphology was a better predictor of most parameters, including whether a species was more likely to exceed their cADL during a dive and the ratio of dive time to cADL. This suggests that benthic and mesopelagic divers are more likely to be foraging at their physiological capacity. For species operating near their physiological capacity (regularly exceeding their cADL), the ability to switch strategies is limited as the cost of foraging deeper and longer is disproportionally high, unless it is accompanied by physiological adaptations. It is proposed that some otariids may not have the ability to switch foraging strategies and so be unable adapt to a changing oceanic ecosystem.

## Teaser

Rapid environmental change may require fur seals and sea lions to make longer or deeper dives to find resources. A systematic review of diving behaviour and physiology revealed that a foraging strategy (epipelagic vs mesopelagic or benthic) was more important in predicting dive behaviour than morphology (fur seal vs sea lion).

## Introduction

Globally, loss of species in marine environments has been slower than in terrestrial systems, but it now appears to be accelerating rapidly (McCauley *et al.* 2015), with increasing consequences predicted for many trophic levels including top predators (Schumann *et al.*, 2013). Humans have profoundly decreased the abundance of many marine fauna and reshaped the genetic structure of a number of marine animal populations through over- and selective harvesting, respectively (McCauley *et al.*, 2015), as well as fundamentally changing the structure of many marine communities (Vergés *et al.*, 2014). Climate change is exacerbating these effects with distribution ranges shifting markedly as temperature regimes alter (Hazen *et al.*, 2012; Brown *et al.*, 2015; Deutsch*et al.*, 2015). These changes raise profound implications for top predators that may have to adapt foraging strategies to cope with shifting prey resources (Bakun *et al.*, 2015; Sydeman *et al.*, 2015; Carroll *et al.*, 2016).

In general, predator foraging strategies account for the predictability and availability of food resources (Sigler *et al.*, 2009; Kuhn 2011; Arthur *et al.*, 2016). While most predators have sufficient inherent flexibility in their foraging strategies to adapt to moderate changes in their prey base (Grémillet and Charmantier, 2010), this capacity is limited by behavioural and physiological capacity. Air-breathing diving marine predators, such as marine mammals, face additional constraints imposed by their physiology and morphology (Schreer and Kovacs, 1997; McCafferty *et al.*, 1998; Rosen *et al.*, 2007). In the face of environmental uncertainty, there is significant potential for the foraging efforts and associated energetic demands of foraging marine predators to increase, particularly in relation to increased search or travel time (Kuhn 2011; Bestley *et al.*, 2015). This is likely to be most severe for the smaller marine mammals such as female otariids (fur seals and sea lions), as adult females are small [30–120 kg except Steller sea lions (*Eumetopias jubata*)], and central place foragers while rearing offspring, and under suboptimal conditions must increase both individual dive duration and trip length (Womble *et al.*, 2009; Staniland *et al.*, 2010; Lowther *et al.*, 2011). Otariids inhabit temperate coastal waters of every continent and are semi-aquatic, high-trophic-level predators. Foraging strategies used by otariids are predominantly pelagic (epipelagic or mesopelagic) or benthic (Costa *et al.*, 2001; Costa *et al.*, 2004; Gallagher *et al.*, 2015). Otariids that forage in the epipelagic zone are typically found in open water (0–400 m) near the continental slope or off the shelf where small schooling prey (crustacea, fish and squid) tend to occur in dense patches that are spatially dynamic (Boyd and Murray, 2001; Harcourt *et al.*, 2002; Robson *et al.*, 2004). Otariids that forage benthically generally feed on larger prey that occur in lower densities but are more evenly distributed over the continental shelf in waters often, though not always, less than 200 m (Littnan and Arnould, 2007; Sigler *et al.*, 2009; Lowther *et al.*, 2011). When otariids forage in the epipelagic zone, foraging dives are short (typically less than 2 min) and shallow (less than 80 m) and are performed less than a third of the time that seals are at sea (e.g. Boyd and Duck, 1991; Trillmich and Kooyman, 2001; Beauplet *et al.*, 2004). Benthic and mesopelagic foraging strategies are usually associated with longer dive durations, greater depths and a higher proportion of time at sea spent diving (TASD) (e.g. Costa and Gales 2000; Fowler *et al.*, 2007; Spence-Bailey *et al.*, 2007), much of which is spent at the bottom of the dive. As a consequence, benthic and mesopelagic divers are more likely to push their physiological limits than their pelagic diving counterparts (Chilvers *et al.*, 2006). Benthic and mesopelagic divers are predicted to spend more time underwater during dives than epipelagic divers, potentially remaining beyond their estimated physiological capacity (Costa *et al.*, 2004; Arnould and Costa 2006; Gallagher *et al.*, 2015).

The aerobic dive limit (ADL) is a measure of the duration of a dive during which an animal uses only aerobic metabolism (Davis 2014, 2019). Since Kooyman *et al.*, (1980) introduced the concept of ADL, it has been used throughout the literature as a measure of the physiological capacity of diving animals. When animals dive beyond their ADL, they are forced to use the less efficient anaerobic metabolism, which results in an accumulation of blood lactate (Kooyman *et al.*, 1983). Lactic acid build-up generally results in a less efficient foraging cycle due to the disproportionate increase in surface durations between dives (Kooyman *et al.*, 1980; Davis and Kanatous, 1999; Davis and Williams, 2012). Measuring the ADL directly through post-dive circulating lactic acid concentrations is a difficult task and to date it has only been measured in two phocids, the Weddell seal (*Leptonychotes weddelli*) (Kooyman *et al.*, 1980) and the Baikal seal (*Phoca sibirica*) (Ponganis *et al.*, 1997c), one otariid, the California sea lion (*Zalophus californianus*) (Ponganis *et al.*, 1997b), and one diving bird, the emperor penguin (*Aptenodytes forsteri*) (Ponganis *et al.*, 1997a). Instead, an estimate of ADL is generally used, which is calculated from total available body oxygen stores and the rate at which these are theoretically depleted by metabolic processes (Costa *et al.*, 2001). This is the calculated ADL (cADL) and represents the time that an air-breathing animal can theoretically dive relying solely on aerobic metabolism (Costa *et al.*, 2001). Although it is unlikely that there is a hard ‘switch’ between aerobic and anaerobic metabolism, cADL provides a standardized comparative measure of the aerobic diving capacity of marine mammals (Gerlinsky *et al.*, 2013). A limitation of using cADL is that it can only be as good as both the measures of metabolic rate and of O_2_ stores for the different species (Butler, 2006), which may change seasonally (Villegas-Amtmann and Costa 2010).

Otariids notably have shorter dive durations and shallower dive depths than other similarly sized air-breathing diving mammals, a pattern that may be a product of physiological constraints related to their aerobic capacity that may explain which prey they target (Schreer *et al.*, 2001). Within otariids, previous research suggested that the relatively small fur seals exceed their cADLs in 2–10% of dives (Gentry and Kooyman, 1986; Boyd and Croxall, 1996), while the larger sea lions may exceed their cADL in 40–60% of dives (Chilvers *et al.*, 2006; Fowler *et al.*, 2007). The body size both empirically and theoretically has a direct influence on diving capacity as metabolic rate scales to body mass^0.75^ while oxygen stores scale with body mass^1.0^. This means that simply due to allometry, larger animals will have a lower mass-specific metabolism for a relatively constant proportion of oxygen storage capacity (Ponganis, 2015) and so larger animals can dive longer and deeper than small ones based on the body size alone (i.e. dive ability scales with body mass^1.25^) (Boyd, 2002). Furthermore, fur seals and sea lions differ in thermoregulatory strategies with sea lions primarily relying on blubber for insulation (Liwanag *et al.*, 2012b), while fur seals use layers of fur to stay warm (Liwanag *et al.*, 2012a). Blubber is relatively incompressible and so maintains a more effective insulative capacity at depth compared to the fur seals’ double fur layer, which relies on air bubbles trapped in the underfur for insulation (Liwanag *et al.*, 2012b).

However, Costa *et al.*, (2004) hypothesized that the foraging strategy (benthic and mesopelagic vs epipelagic) was the main driver of relative foraging effort rather than morphology (sea lion vs fur seal) *per se*. They reported that benthic and mesopelagic divers were far more likely to exceed their cADL, spend more time at sea diving and have greater dive durations (Costa *et al.*, 2004; Gallagher *et al.*, 2015). They concluded that sea lions, as a result of being primarily benthic and/or mesopelagic divers, are likely operating near or at their physiological maximum (Chilvers *et al.*, 2006; Fowler *et al.*, 2007; Villegas-Amtmann and Costa, 2010). Diving for periods longer than the cADL is correlated with reduced foraging efficiency (Boyd *et al.*, 1997) and may represent a limitation in their scope for behavioural adaptation to changing ecosystems. The proportion of dives that exceed an animal’s cADL can be used as a measure of the species’ dive performance.

Foraging efficiency is generally considered in terms of the proportion of the dive cycle spent actively pursuing prey. Hence, for marine mammals, foraging efficiency increases by maximizing their bottom time (Austin *et al.*, 2006), and this is directly influenced by vertical distance travelled (Kramer, 1988). Regardless of whether otariids use an epipelagic, mesopelagic or benthic foraging strategy, environmental changes may require them to switch to deeper or longer dives. When seals are compelled to repeatedly dive for longer periods (e.g. if in a rich prey patch), they may have the ability to accumulate oxygen debt and replenish their stores later (Horning, 2012). The ability to increase their foraging effort without impacting their foraging efficiency (through increased reliance on anaerobic metabolism) is limited by their physiological capacity. An alternate strategy is to alter their overall foraging strategy rather than increase foraging effort under challenging prey conditions. While this theoretically makes sense, there is very little evidence that fur seals switch foraging strategies during periods of low prey abundance (Boyd *et al.*, 1994) and clear evidence that at least some benthic foragers continue to use the same foraging habitat even when the fish guild structure changes significantly (Lowther *et al.*, 2013). This suggests that some species may be physiologically constrained in their capacity to respond to environmental challenges as identified in Costa *et al.*, (2004) and Arnould and Costa (2006). These original studies only looked at a subset of extant otariids and were based on the limited evidence available produced at that time. Diving studies on marine mammals inherently suffer from small sample sizes and become more robust as sample sizes increase (Sequeira *et al.*, 2019). Over the past 15 years, numerous new studies of dive behaviour using larger numbers of animals and more species have appeared. This provides an opportunity to revisit the earlier forecasts of Costa and colleagues. Therefore, the aim of this review is to (i) summarize physiological and diving parameters for otariid species with the most up-to-date literature, (ii) revisit the approach devised by Costa *et al.*, (2004) and Arnould and Costa (2006) by comparing and contrasting the diving characteristics (dive performance and foraging efficiency) of fur seals and sea lions as epipelagic vs mesopelagic and benthic divers and (iii) identify those species that may be operating at or near their physiological maximum and the implications of this in a changing world.

## Methods

The family Otariidae (colloquially known as ‘otariids’) consists of nine extant species of fur seal (Arctocephalinae) with one subspecies and six species of sea lion (Otariinae) (Wolf *et al.*, 2007). Despite no clear phylogenetic distinction between sea lions and fur seals (Berta and Churchill, 2012), we differentiate and compare these groups within this study because of their distinct morphological differences. The primary distinction between the two groups is their size and thermoregulation strategy, where the smaller fur seals rely more on their fur while the larger sea lions rely more on blubber for insulation (Berta *et al.*, 2005). Using an array of search platforms (Google Scholar and Macquarie University Library) and databases (Wiley Online and Elsevier), we collected information from 16 species of lactating female otariid (where available). Physiological parameters were collected first on as many species as possible. Physiological parameters included total body oxygen stores (TBOS), diving or field metabolic rate (DMR or FMR), cADL and average body mass. Behavioural diving parameters were then collected from published sources for these same species when possible. Behavioural diving parameters included average and maximum dive duration and depth, percentage of TASD and post-dive surface interval (Tables 1 and 2). Not all of the required parameters were available for all 16 species and in some cases the value was inferred from a species matched from phylogenetic relationships informed by morphology (Churchill *et al.*, 2014) and assumed to be equivalent (Costa *et al.*, 2004).

**Table 1:** Summary diving parameters for 15 species of predominantly epipelagic diving otariids

Common name	Scientific name	Distribution (latitude)		*N*	Mass	Depth	Max depth	Duration	Max duration	TASD	Dives per trip	Trip duration	Colony duration
Pelagic		Min	Max		kg	m	m	min	min	%	*N*	Days	Days
Antarctic fur seal^1^^,^^2^^,^^3^	*A. gazella*	−65	−50	49	32.7	29.2	122.9	1.3	3.7	29.0	1217	2.9	1.0
Subantarctic fur seal^1^^,^^3^^,^^4^	*A. tropicalis*	−65	−50	47	43.1	20.4	127.6	1.6	3.4	23.2	1208	1.7	1.2
Northern fur seal^2^^,^^5^^,^^6^	*Callorhinus ursinus*	30	65	33	39.3	26.3	71.5	1.7	4.4	57.9	1725^*^	2.8	1.2
Galapagos fur seal^6^^,^^7^^,^^8^^,^^9^	*A. galapagoensis*	−2	1	21	28.8	31.4	131.0	1.7	5.4	23.7	1049	1.2	1.0
New Zealand fur seal^10^^,^^11^	*A. forsteri*	−50	−35	26	42.4	41.5	180.0	2.7	9.3	37.6	1046	6.8	2.1
Guadalupe fur seal^12^	*A. philippii townsendi*	27	40	1	52.5	15.0	27.1	2.6	18.0	45.5	1465	20.2	3.6
Cape fur seal^13^	*A. pusillus*	−15	−35	6	75.0	45.0	197.5	2.1	7.0	57.8	NA	NA	NA
South American fur seal^14^^,^^15^	*A. australis*	−20	−55	3	48.5	43.0	113.7	2.8	5.3	20.8	NA	2.3	1.9
California sea lion^16^^,^^17^^,^^18^^,^^19^	*Z. californianus*	20	50	11	82.4	75.0	143.2	2.8	7.7	38.5	1270^**^	2.7	1.0
Juan Fernandez fur seal^20^	*A. philippii*	33	34	18	48.0	12.3	66.1	0.8	3.4	2.6	202	12.3	5.3
Mixed													
Galapagos sea lion^21^^,^^22^	*Zalophus wollebaeki*	−2	1	2	78.0	146.1	429.7	2.6	9.0	65.7	2130	0.5	0.4
Southern sea lion^23^^,^^24^^,^^25^	*Otaria byronia*	0	−55	16	126.0	60.0	47.0	2.7	6.1	44.2	672	2.8	1.6

^*^Average number of dives per bout × average number of bouts per foraging trip.

^**^Calculated from average dives per hour × hours per foraging trip.

1. Luque *et al.*, (2008); 2. Jeanniard-du-Dot *et al.*, (2016); 3. Bailleul *et al.*, (2005); 4. Georges *et al.*, (2000b); 5. average of pelagic divers: Kuhn (2011); 6. Skinner *et al.*, (2012); 7. Horning and Trillmich (1997); 8. Villegas-Amtmann *et al.*, (2013); 9. Gentry and Kooyman (1986); 10. Page *et al.*, (2005); 11. Harcourt *et al.*, (2002); 12. Gallo-Reynoso *et al.*, (2008); 13. Kooyman and Gentry (1986); 14. average: Trillmich *et al.*, (1986); 15. Thompson *et al.*, (2003); 16. Kuhn and Costa (2014); 17. average deep divers: McHuron *et al.*, (2018); 18. Strategy 1: Kuhn and Costa (2014); McHuron (2016); 19. Feldkamp *et al.*, (1988); 20. Francis *et al.*, (1998); 21. average shallow divers: Villegas-Amtmann and Costa (2010); 22. Villegas-Amtmann *et al.*, (2008); 23. Werner and Campagna (1995); 24. average: Costa and Gentry (1986); 25. Baylis *et al.*, (2015a).

Information has been collated from published reports of diving parameters for species listed. A southern distribution is represented by negative latitude and is an estimate only. *N* is the sample size, mass is the average mass of animals studied, depth and duration are the average typical depths and durations of otariids dive, max depth and max duration are the maximum depth and maximum duration recorded by any otariid and TASD is the percentage time at sea spent diving.

**Table 2:** Summary diving parameters for eight benthic and mesopelagic divers

Common name	Scientific name	Distribution (latitude)		*N*	Mass	Depth	Max depth	Duration	Max duration	TASD	Dives per trip	Trip duration	Colony duration
Benthic		Min	Max		kg	m	m	min	min	%			
Australian fur seal^1^	*Arctocephalus pusillus doriferus*	−35	−45	13	77.7	58.0	89.1	2.9	6.7	40.7	849	4.3	1.9
Southern sea lion^2^^,^^3^^,^^4^	*O. byronia*	0	−55	4	126.0	99.0	158.0	2.6	6.1	30.5	538	3.2	1.6
Australian sea lion^5^	*Neophoca cinerea*	−25	−35	29	69.3	62.3	83.1	3.3	4.1	57.3	688	2.1	2.0
New Zealand sea lion^6^	*Phocarctos hookeri*	−40	−50	11	112.3	124.0	353.0	3.4	8.3	44.9	831	4.4	2.1
Galapagos sea lion^7^^,^^8^^,^^9^	*Z. wollebaeki*	−2	1	10	78.0	103.0	571	4.9	9.6	53.9	1767	1.1	0.5
Steller sea lion^10^	*Eumetopias jubatus*	45	60	11	194.0	25.3	236.0	1.6	16.0	22.0	280	0.87	0.87
Normally pelagic													
Northern fur seal^11^^,^^12^	*C. ursinus*	30	65	33	36.8	79.5	205.0	3.2	5.4	33.8	1725	2.8	1.2
California sea lion^13^^,^^14^^,^^15^	*Z. californianus*	20	50	37	83.2	164.7	350..9	3.9	7.7	42.7	1326	4.3	1.3

1. Arnould and Hindell (2001); 2. Costa and Gales (2003); 3. Werner and Campagna (1995); 4. Costa and Gales (2000); Baylis *et al.*, (2015b); 5. average: Costa and Gales (2003); 6. Costa and Gales (2000); 7. average: Villegas-Amtmann and Costa (2010); 8. Villegas-Amtmann *et al.*, (2013); 9. Villegas-Amtmann *et al.*, (2008); 10. Rehberg *et al.*, (2009); 11. Ponganis *et al.*, (1992); 12. average benthic dives: Kuhn (2011); 13. average mixed divers: McHuron *et al.*, (2018); 14. average strategies 2 and 3: McHuron *et al.*, (2016); 15. Feldkamp *et al.*, (1988).

Information has been collated from published reports of diving parameters for species listed. A southern distribution is represented by negative latitude and is an estimate only. *N* is the sample size, mass is the average mass of animals studied, depth and duration are the average typical depths and durations otariids dive, max depth and duration are the maximum depth and duration recorded by any otariid and TASD is the percentage time at sea spent diving.

Values of cADL are only reported for those species where either it had been previously calculated and published or only one required parameter was missing (either TBOS or DMR—see below). This enabled us to report or estimate the cADL for females of six fur seal and six sea lion species. We focussed on females as these were most likely to be physiologically constrained due to their smaller size and the impact that changes in foraging efficiency have on nursing offspring. There was insufficient information to include Guadalupe fur seals (*Arctocephalus philippii townsendi*), South American fur seals (*Arctocephalus australis*), Cape fur seals (*Arctocephalus pusillus pusillus*) or New Zealand fur seals (*Arctocephalus forsteri*) to calculate cADL.

While cADL is only an estimate of actual ADL (Gerlinsky *et al.*, 2013), it remains the best standardized measure of diving abilities available (Butler, 2006). In this paper, cADL values were either taken directly from the literature as the author reported it or calculated from other available physiological data, specifically by dividing TBOS (mL O_2_ kg^−1^) by the DMR (mL O_2_ kg^−1^ min^−1^) of the animal (Costa *et al.*, 2001):

(1) \begin{equation*} \text{cADL (min) = TBOS/DMR} \end{equation*}

cADL and reported cADL differed significantly, and there were no reported cADL values for all species. Therefore, for consistency, only cADL values (those which were derived from the above formula) were used for analyses and reported cADL values are captured in the tables but not .

TBOS are the combined oxygen stores in the lung (usually estimated as lung volume multiplied by the fraction of air in the lungs that is assumed to be oxygen at the start of a dive), blood (calculated from measures of blood volume, haemoglobin concentration and the haemoglobin oxygen binding capacity) and muscle (calculated from estimates of muscle mass, myoglobin concentration and the myoglobin oxygen binding capacity) (Lenfant *et al.*, 1970). Insufficient data were available to provide an estimate of TBOS for Galapagos fur seal (*Arctocephalus galapagoensis*) and South American fur seal (*Arctocephalus australis*) so the estimate for Antarctic fur seal (*Arctocephalus gazella*) was . All estimates were adjusted for mass and are detailed in Table 3.

**Table 3:** Summary physiological parameters for epipelagic divers

Common name	*N*	Mass	TBOS		cADL (min)		FMR (ml O_2_ kg^−1^ min ^−1^)	
		kg	ml O_2_ kg^−1^	Method	Reported	Calculated^±^	Reported	Method
Antarctic fur seal^1^	15	41.9	44.6	Bloods	1.6	1.5	29.6	DLW
Subantarctic fur seal^2^	14	43.1	36.0	Bloods	2.6	2.6	14.1^#^	Estimate from AFS
Northern fur seal^3^^,^^4^	7	34.1	41.0	Bloods	2.6	1.3	19.9	Respirometry
Galapagos fur seal^5^^,^^6^	NA	30.0	62.3	Estimate from AFS	3.3	4.4	14.2	DLW
New Zealand fur seal^7^^τ^	NA	NA	35.7^*^	Estimate from males	NA	2.6	NA	-
Guadalupe fur seal^τ^	NA	NA	NA	-	NA	2.7	NA	-
Cape fur seal^τ^	4	53.0	NA	-	NA	2.9	NA	-
South American fur seal^τ^	NA	NA	NA	-	NA	2.6	NA	-
California sea lion^8^^,^^9^^,^^10^	11	82.4	51.5	Bloods	3.8	2.6	20.1	DLW
Juan Fernandez fur seal^11^	10	48.0	46.2^**^	Bloods		2.6	17.6^#^	Estimate from GFS
Normally benthic								
Galapagos sea lion^12^^,^^13^	2	78.0	62.8	Bloods	NA	3.8	16.5	
Southern sea lion^14^^,^^15^	10	101.7	46.0	Bloods	2.4	2.2	21.2	Respirometry

± ^±^Calculated in this review as TBOS/FMR.

^*^Estimate using 22.6 (ml O2)/0.1 (l) = 226 (ml/l of blood) × 20 BV (l)/106.4, BM (kg) = 42.5 ml O_2_/kg where 20 BV (l) is taken from California sea lion;

^**^Estimate from 19.2 (ml O2)/0.1 (l) = 192 (ml/l of blood) × 13.2 BV (l)/48 BM (kg) = 46.2 ml O2/kg where 13.2 BV (l) is taken from Antarctic fur seal;

1. Costa *et al.*, (2001); 2. Verrier *et al.*, (2011); 3. Shero *et al.*, (2012); 4. Rosen *et al.*, (2017); 5. Trillmich and Kooyman (2001); 6. Horning and Mellish (2012); 7. Wells (1978); 8. Neises *et al.*, (2017); 9. Weise and Costa (2007); 10. average of mixed divers: McHuron *et al.*, (2018); 11. Sepúlveda *et al.*, (1999); 12. average shallow divers: Villegas-Amtmann and Costa (2010); 13. Villegas-Amtmann *et al.*, (2017); 14. Hückstädt *et al.*, (2016); 15. Dassis *et al.*, (2012);

τ ^τ^Not included in formal statistical tests as not enough information available.

Information has been collated from published reports of diving parameters for species listed. *N* is the sample size, mass is the average mass of animals studied, TBOS is the total body oxygen stores, which have either been estimated from other species (Estimate) or from blood samples (Bloods), cADL is the calculated ADL and FMR is the field metabolic rate, which has either been estimated from other species (Estimate), from doubly labelled water (DLW) or from respirometry (Respirometry).

DMR is a measure of the energy that is expended during submerged activity. For this review, we prioritized DMR data that were directly measured via respirometry, and where this was not available, we values estimated via doubly labelled water in the field (or FMR). Values estimated via respirometry are generally regarded as the ‘gold-standard’ of energy expenditure measurements and take into account only the energy expended during the dive (Rosen *et al.*, 2016). FMR is an estimate of energy expenditure over an entire foraging trip, creating difficulties in extracting activity-specific measures of energy expenditure (Costa *et al.*, 1989; Dalton *et al.*, 2014). To more accurately capture diving effort, only at-sea FMR (i.e. excluding measures incorporating on-land periods) was as an estimate of DMR. Either method of estimating diving metabolism may lead to over- or under-estimates of the true cADL; thus, we use both to allow for comparisons among species.

Where possible, estimates of DMR were taken from published material and, when necessary, converted into ml O_2_ min^−1^ kg^−1^. Any estimates of DMR that were reported as W kg^−1^ were converted into ml O_2_ min^−1^ kg^−1^ using the following calculation:

(4) \begin{align*} \quad DMR (ml \ O_2 \ min^{-1} kg^{-1}) \quad\quad\quad\quad\quad\quad\quad\quad&\nonumber\\= (FMR (W \ kg^{-1})\times 0.0143)/5.05\times1000 \end{align*}

DMR was not available for two species [Juan Fernandez fur seals (*Arctocephalus philippii philippii*), Subantarctic fur seals (*Arctocephalus tropicalis*)] and, for these, DMR was calculated from an estimate from a similar species (Table 3) and adjusted for the body mass of the target group:

(5) \begin{align*} DMR_{target} (ml \ O_2 \ min^{-1} kg^{-1}) \quad\quad\quad\quad\quad\quad\quad\quad&\nonumber\\= DMR_{similar} (ml \ O_2 \ min^{-1} kg^{-1})\nonumber\\ \times BM_{similar}/ BM_{target}\quad\quad\quad\quad \end{align*}

where DMR = diving metabolic rate, BM = body mass, target = target species and similar = similar species.

Diving behavioural data were taken from published reports of wild otariid foraging. The average and maximum dive duration, the average and maximum dive depth, and proportion of time at sea diving (TASD), the number of seals it was calculated for and the standard deviation for each value were extracted for each species where it was available. TASD was not always recorded, but a diving rate (dives per hour) was available. We the following to estimate TASD from other dive parameters:

(6) \begin{align*} \quad TASD = (dive rate (dives/hr)\quad\quad\quad\quad\quad\quad\quad\quad\quad\nonumber\\ \times mean \ dive \ duration \ (min))/60\times100 \end{align*}

where standard deviation was not available a crude estimate was made as

(7) \begin{align*} SD= (\max-\min)/3.5 \end{align*}

Species were categorized as benthic and mesopelagic or epipelagic divers based on their primary mode of foraging (Costa *et al.*, 2004; Arnould and Costa 2006). Species that foraged primarily on demersal prey on the benthos were regarded as benthic foragers. Those that foraged in the deep pelagic zone (below 200 m) were classified as mesopelagic foragers and those that foraged in the upper pelagic zone (above 200 m), tracking their prey through migratory patterns, were classified as epipelagic foragers. Where research found multiple strategies within a single species, the information for each type of foraging mode was captured and analysed separately.

## Statistical analysis

A key goal of the study was to estimate how often animals dived beyond their cADL. This cannot be determined from point estimates of dive duration (e.g. mean or maximum), so we simulated a series of dive durations for the 13 species—6 fur seals and 7 sea lions—with complete dive parameters available from the literature. Simulated distributions were created with the mean and max dive duration and the number of seals from which the parameters were derived. We simulated diving durations using negative binomial distributions using the MASS package in R (Ripley *et al.*, 2013) and then calculated the percentage of those dives that exceeded the cADL. We first estimated theta using $\mu$ = mean dive duration, *y* = average number of dives on a single foraging trip, df = number of seals − 1, then we simulated the dive distribution of a given species using the function *rnegbin* from the MASS package with 20 000 simulations, with the estimated theta and the specified limits of lower = 0 and upper = max dive time. We ran the simulation for the estimated number of foraging trips conducted on average each year for each species dive behaviour group. Average number of foraging trips was estimated from average duration spent at sea (days) and average colony duration (days). For example, the average number of dives that exceed the cADL for the Australian fur seal is estimated by first calculating theta using an average dive time of 2.9 min for an average of 849 dives per foraging trip from 13 seals. Theta is then to simulate the dive distribution for a given foraging trip with a maximum dive duration of 6.7 min, where the number of dives exceeding the cADL of 2.5 min. The simulation is repeated 59 times, the average number of foraging trips conducted per year. The final value is the mean value of proportion of dives that exceed the cADL of the simulated foraging trips.

All analyses were conducted in R and, before any parametric testing was conducted, all relevant assumptions (i.e. homogeneity of variances and normality) were tested and met. The summarized data for each species were rather than individual values as the data for individuals were rarely available, and it has been shown that the conclusions and effect sizes made from summary data are very similar to those made with individual data (Steinberg *et al.*, 1997; Tudur Smith *et al.*, 2016). Two-way analysis of variance (ANOVA) for summary data (Cohen 2002) were to look for statistical differences and interactions between and within foraging mode (benthic and meso vs. epipelagic) and morphology group (sea lion vs. fur seal). To implement the ANOVAs, the mean, standard deviation and sample size were included in the formula and implemented using the function ind.twoway.second() in the R package rpshychi (Okumura and Okumura 2012).

Pearson’s correlation tests were to examine the relationship between all the response variables (mass, depth, duration, TASD, TBOS, DMR, cADL, dive performance and percentage of dives that exceed the cADL). Dive performance is measured as the ratio of mean dive duration to cADL (Arnould and Costa 2006).

**Table 4:** Summary physiological parameters for eight benthic and mesopelagic divers

Common name	*N*	Mass	TBOS		cADL (min)		FMR (ml O_2_ kg^−1^ min ^−1^)	
		kg	ml O_2_ kg^−1^	Method	Reported	Calculated^±^	Reported	Method
Australian fur seal^1^	1	71.2	46.0	Bloods	2.4	3.0	15.2	DLW
Southern sea lion^2^^,^^3^	10	129.9	34.0	Bloods	1.9	1.6	21.2	Respirometry
Australian sea lion^4^^,^^5^	4	66.4	47.0	Bloods	2.3	4.2	11.2	DLW
New Zealand sea lion^4^^,^^6^	11	112.4	47.4	Bloods	2.3	2.3	20.3	DLW
Galapagos sea lion^7^^,^^8^	7	78.6	62.7	Bloods	NA	4.0	15.6	DLW
Steller sea lion^9^	4	193.0	35.9	Bloods	3.0	2.8	12.6	Respirometry
Mixed								
Northern fur seal^10^^,^^11^	7	36.9	41.0	Bloods	2.6	2.2	18.4	Respirometry
California sea lion^12^	4	86.7	51.5	Bloods	3.8	2.7	19.2	DLW

± ^±^Calculated in this review as TBOS/FMR.

1. Spence-Bailey *et al.*, (2007); 2. Dassis *et al.*, (2012); 3. Hückstädt *et al.*, (2016); 4. Costa *et al.*, (2001); 5. average: Ladds *et al.*, (2016); 6. Costa and Gales (2000); 7. average deep and bottom: Villegas-Amtmann and Costa (2010) 8. Villegas-Amtmann *et al.*, (2017); 9. average: Gerlinsky *et al.*, (2013); 10. Shero *et al.*, (2012); 11. Rosen *et al.*, (2017); 12. average of deep divers: McHuron *et al.*, (2018)

Information has been collated from published reports of diving parameters for species listed. *N* is the sample size, mass is the average mass of animals studied, TBOS is the total body oxygen stores, which have either been estimated from other species (Estimate) or from blood samples (Bloods), cADL is the calculated ADL and FMR is the field metabolic rate, which has either been estimated from other species (Estimate), from doubly labelled water (DLW) or from respirometry (Respirometry).

**Table 5:** Averages (±SD) of diving and physiological parameters for family and foraging strategy and results of two-way ANOVAs

Variable	Foraging strategy	Family		ANOVA results			95% CI	
		Fur seal	Sea lion	Test	*F*	η^2^	Lower	Upper
Depth (m) *N* = 19	Benthic and mesopelagic	68.8 (±22.6)	96.4 (±25.4)	Interaction	1.324	0.000	0.000	0.028
				Family	84.85	0.204	0.132	0.277
	Pelagic	29.3 (±16.6)	93.7 (±63.4)	Foraging strategy	106.97	0.244	0.169	0.318
Duration (min) *N* = 19	Benthic and mesopelagic	3.0 (±0.8)	3.0 (±0.5)	Interaction	20.34	0.058	0.019	0.122
				Family	21.25	0.060	0.020	0.116
	Pelagic	1.9 (±0.9)	2.7 (±0.7)	Foraging strategy	72.38	0.179	0.111	0.251
TASD (%) *N* = 19	Benthic and mesopelagic	37.3 (±7.8)	39.9 (±8.1)	Interaction	49.41	0.130	0.070	0.198
				Family	90.46	0.215	0.142	0.288
	Pelagic	33.1 (±7.1)	50.9 (±5.9)	Foraging strategy	10.01	0.029	0.004	0.073
Total oxygen stores (ml O_2_ kg^−1^) *N* = 16	Benthic and mesopelagic	43.5 (±5.0)	46.4 (±7.2)	Interaction	6.66	0.020	0.001	0.058
				Family	34.72	0.095	0.043	0.158
	Pelagic	46.0 (±5.6)	53.4 (±8.8)	Foraging strategy	29.49	0.082	0.034	0.142
DMR (ml O_2_ min^−1^ kg^−1^) *N* = 12	Benthic and mesopelagic	16.8 (±4.6)	16.7 (±3.1)	Interaction	1.78	0.005	0	0.032
				Family	1.17	0.004	0	0.027
	Pelagic	18.0 (±4.5)	19.3 (±4.0)	Foraging strategy	14.18	0.041	0.009	0.090
cADL (min) *N* = 19	Benthic and mesopelagic	2.6 (±0.2)	2.9 (±0.2)	Interaction	10.58	0.031	0.005	0.076
				Family	131.47	0.284	0.207	0.357
	Pelagic	2.7 (±0.1)	2.8 (±0.2)	Foraging strategy	1.87	0.006	0.0	0.032
Ratio (cADL/dive time) *N* = 19	Benthic and mesopelagic	1.2 (±0.001)	1.1 (±0.4)	Interaction	6.86	0.020	0.001	0.059
				Family	0.0	0.0	0.0	0.0
	Pelagic	0.7 (±0.3)	1.0 (±0.2)	Foraging strategy	80.10	0.195	0.124	0.267
Dives exceeding cADL (%) *N* = 19	Benthic and mesopelagic	55.6 (±1.4)	35.8 (±1.7)	Interaction	2822.6	0.895	0.877	0.909
				Family	1314.8	0.799	0.764	0.825
	Pelagic	25.8 (±1.5)	29.5 (±1.2)	Foraging strategy	6666.9	0.953	0.944	0.959

η^2^ is the estimate of the effect size of the difference and 95% CI is the confidence interval of the estimate.

**Figure 1:**
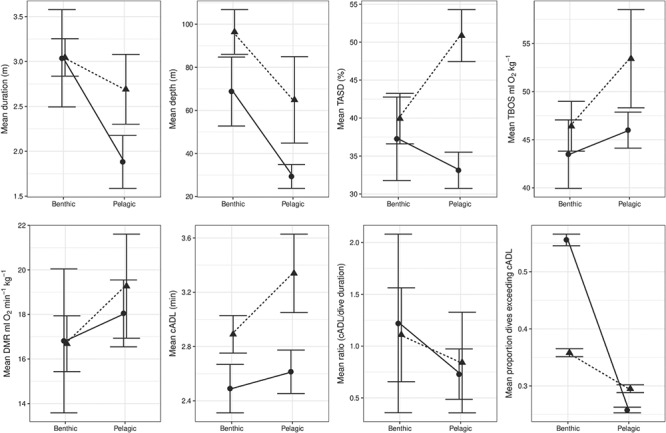
Interaction plots for the means ± SE of diving (depth, duration, TASD) and physiological (TBOS, DMR, cADL, ratio and % dives exceeding the cADL) variables for foraging strategy (benthic and mesopelagic vs epipelagic) and morphology (fur seal—circles, sea lion—triangles) for female otariids

**Figure 2:**
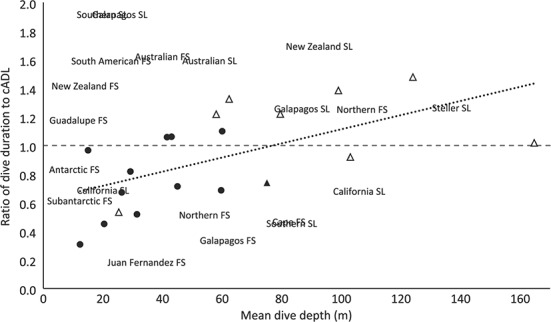
Relationship between the ratio of cADL to mean dive duration and mean dive depth for 7 female sea lions (squares) and 12 female fur seal (circles). Filled shapes represent pelagic divers and open shapes are benthic divers. Values less than one for the ratio of dive duration to cADL indicate that seals are diving on average shorter than their cADL; values greater than one indicate that seals have average dive durations greater than their cADL. Regression line shown in dotted line; relationship is estimated from least-squares regression.

**Figure 3:**
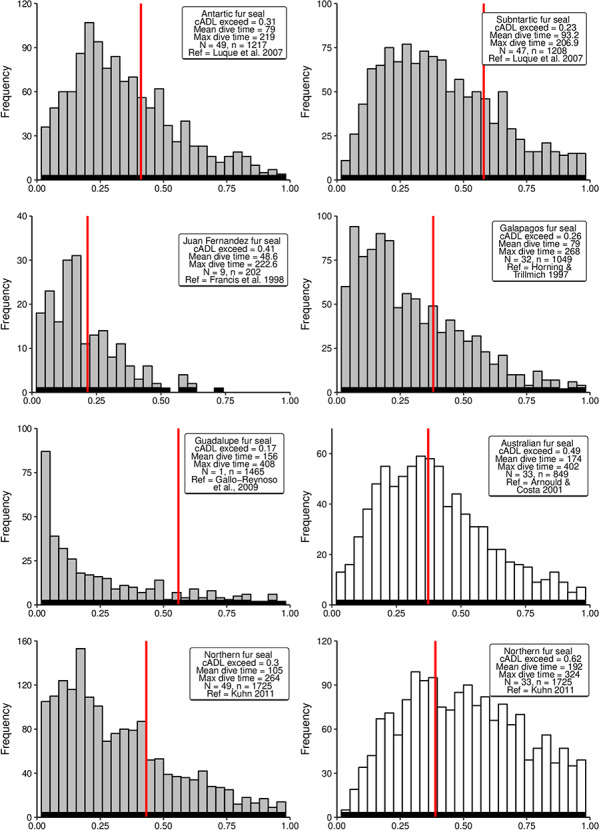
Distributions of simulated diving durations from a negative binomial distribution for seven fur seal species. The *x*-axis is the scaled dive duration for each sepecies (dive duation/maximum dive duration). Grey histograms represent pelagic divers; white histograms represent benthic divers; red line represents cADL for each species. *N* = number of seals; *n* = number of dives.

## Results

Values to calculate the following results are in Tables 1–4 and the summary statistics derived related to the following results are in Table 5. cADL varied primarily by whether the species was a sea lion ($\overline{x}=$2.9, SE = 0.30 min) or fur seal ($\overline{x}=$2.7, SE = 0.22 min) (Table 5). Some variation in cADL could be explained by foraging strategy (epipelagic vs benthic or mesopelagic) (Table 5) where epipelagic foragers had a longer average cADL than benthic and mesopelagic divers ($\overline{x}=$2.9, SE = 0.31 and $\overline{x}=$2.7, SE = 0.22 min, respectively; Fig. 1). No variation could be explained by the interaction between the two (Table 5).

TBOS varied both by morphology group (Table 5), with sea lions having larger TBOS than fur seals ($\overline{x}=$48.74, SE = 3.34 ml O_2_ min kg^0.75^ vs $\overline{x}=$45.54, SE = 3.10 ml O_2_ min kg^0.75^) and by foraging strategy with epipelagic divers having larger TBOS ($\overline{x}=$47.86, SE = 3.14 ml O_2_ min kg^0.75^ vs $\overline{x}=$45.68, SE = 3.22 ml O_2_ min kg^0.75^). No variation could be explained by the interaction between the two (Table **5**). A small amount of variation in DMR could be explained by foraging strategy (Table 5), where epipelagic divers had higher DMR’s ($\overline{x}$= 18.35, SE = 1.35 ml O_2_ min^−1^ kg^−1^ vs benthic and mesopelagic $\overline{x}$= 16.72, SE = 1.29 ml O_2_ min^−1^ kg^−1^).

Both foraging strategy, morphology and their interaction accounted for variation in dive depth and duration (Table 5). Morphology accounted for a large amount of the variation in depth, while foraging strategy accounted for a large amount of variation in duration (Table 5). There was a large effect of morphology on TASD and a small interaction effect of morphology and foraging strategy (Table 5). On average sea lions dived deeper (sea lion $\overline{x}$ = 95.49, SE = 14.89 m vs fur seal $\overline{x}$ = 36.51, SE = 5.99 m), for longer (sea lion $\overline{x}$= 2.93, SE = 0.23 min vs fur seal $\overline{x}$ = 2.09, SE = 0.24 min) and spent a greater proportion of their time at sea diving ($\overline{x}$ = 43.57, SE = 4.69% vs $\overline{x}$ = 33.87, SE = 4.98%) than fur seals. Dive performance measured by the ratio of cADL and average dive duration was influenced by foraging strategy (Table 5). The ratio was larger for benthic and mesopelagic divers (benthic and mesopelagic $\overline{x}$= 1.13, SE = 0.11 vs epipelagic $\overline{x}$ = 0.76, SE = 0.07). The proportion of dives that exceed the cADL varied largely by foraging strategy (Table 5) where benthic and mesopelagic divers were far more likely to exceed the cADL (benthic and mesopelagic $\overline{x}$ = 40.75., SE = 6.16% vs epipelagic $\overline{x}$ = 26.90, SE = 3.13%).

There was no relationship between body mass and dive depth or dive duration (Pearson’s correlation test: *P* > 0.05 for both tests). There was no significant correlation between TBOS and dive depth or duration (*P >* 0.05). A positive correlation was found between dive performance (the ratio of dive duration to cADL) and dive depth (correlation coefficient = 0.46, *P* = 0.04; Fig. 2). Similarly, there was a positive correlation between percentage of dives that exceed the cADL and dive depth (correlation coefficient = 0.33, *P* = 0.18), where benthic and mesopelagic divers were more likely to exceed their cADL (Fig. 3).

**Figure 4:**
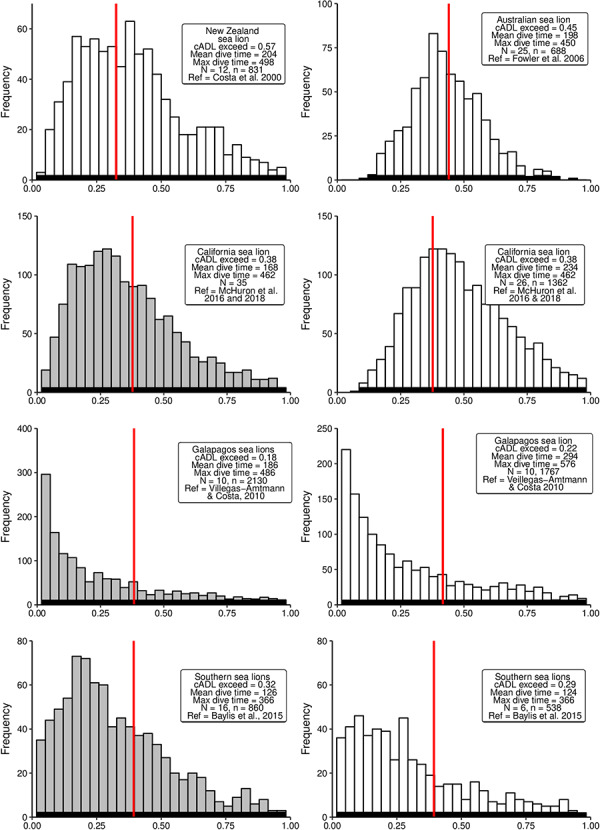
Distributions of simulated diving durations from a negative binomial distribution for six sea lion species. The *x*-axis is the scaled dive duration for each sepecies (dive duation/maximum dive duration). Grey histograms represent pelagic divers; white histograms represent benthic divers; red line represents cADL for each species. *N* = number of seals; *n* = number of dives.

**Figure 5:**
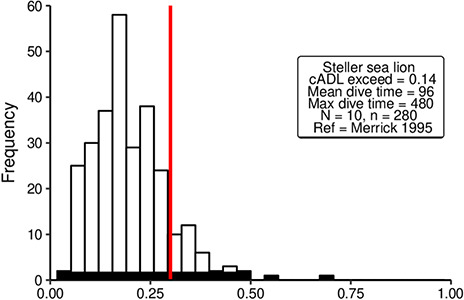
Distributions of simulated diving durations from a negative binomial distribution for Steller sea lions (benthic divers). The *x*-axis is the scaled dive duration for each sepecies (dive duation/maximum dive duration). Red line represents cADL for each species. *N* = number of seals, *n* = number of dives.

Overall, the probability of exceeding the cADL on any given dive was 1.5 times more likely for benthic and mesopelagic diving animals than for an epipelagic diver. Dive durations of epipelagic divers were predominantly less than half the duration of their maximum dive time (Figs 3 and 4). For benthic and mesopelagic diving animals, the distributions were more normally distributed (Figs 3–5). Benthic diving Northern fur seals were most likely to exceed their cADL, diving beyond the estimated threshold on 62% of dives.

### Comparison of multi-strategy animals

There were three species that foraged using both a benthic or mesopelagic strategy and an epipelagic strategy [Northern fur seals (Fig. 3), Southern sea lions and Galapagos sea lions (Fig. 4)]. Many of the benthic dives exceed the cADL for Northern fur seals (62%), while most pelagic and benthic dives were within the cADL for Southern sea lions (68–71%) and Galapagos sea lions (78–82%).

## Discussion

Global changes to ocean ecosystems are predicted to result in range shifts for many otariid prey species (Costa *et al.*, 2004; Bakun *et al.*, 2015). For predators, this means that they may need to increase their foraging effort in order to gain sufficient energy, as failing to meet energetic demands directly impacts survival (Boyd *et al.*, 1994). Diving mammals may be required to forage deeper and for longer (Costa *et al.*, 2004), and/or target larger demersal prey that are predicted to be less influenced by changes in the ocean ecosystem (Perry *et al.*, 2005). It has been hypothesized that species that normally dive within their ADL can increase foraging effort with fewer consequences by drawing on oxygen reserves to pursue prey at depth (Boyd *et al.*, 1994; Costa *et al.*, 2004). However, those species already operating at or near their physiological maximum may not have a similar capacity to increase foraging effort.

By reviewing the physiological (DMR, TBOS and cADL) and behavioural (dive depth, duration and TASD) information of females from every extant otariid, we have shown that foraging mode (benthic and mesopelagic or epipelagic), rather than morphology (fur seal or sea lion), is more indicative of which species operate at or near their physiological maximum. Crucially, we reviewed abilities for females that are not only more likely to be physiologically constrained, but are the limiting sex, directly influencing the size of populations. We found that female otariids that dive using a benthic or mesopelagic strategy forage deeper and longer and exceed their cADL 1.4 times as frequently as do epipelagic divers, regardless of whether they were a fur seal or sea lion. This may make the former more vulnerable to environmental changes that cause prey to move deeper or further offshore as their physiological scope for adaptation to diving deeper and longer is limited (and switching foraging strategies from benthic to epipelagic would not help them find prey). In contrast, as epipelagic divers generally do not currently exceed their cADL, they may have more scope to switch to a benthic foraging strategy; although if they do, the likelihood of exceeding their cADL substantially increases (Northern fur seal—Fig. 3). Therefore, switching foraging modes during periods of low prey abundance is not a risk-free solution to finding additional energy during these times.

Our study confirms that foraging strategy is a better predictor than morphology (fur seal vs sea lion) for identifying species operating near their physiological maximum and accords with earlier theorizing (Costa *et al.*, 2004; Arnould and Costa 2006). We found that foraging strategy accounted for variation in more behavioural parameters than morphology, as either the sole predictor of variation (cADL ratio and depth) or as the interaction between morphology and foraging strategy (dives exceeding cADL, duration and TASD). In fact, morphology could only directly account for variation in one behavioural parameter, depth. Benthic and mesopelagic divers were more likely to exceed their cADL on a given dive, as demonstrated by an average cADL ratio (cADL/dive duration) greater than one. They dived to deeper depths for longer and, despite having relatively low DMR’s, also had smaller TBOS that limited their ability to dive longer within their cADLs (Fig. 1). Deeper dives were related to larger cADL ratios meaning that, as they dived deeper, they were more likely to exceed their cADLs. Benthic diving sea lions were most likely to dive for longer on average than their cADL (Fig. 2), while most of the epipelagic foragers dived on average for durations less or equal to theircADL.

The otariids that are currently operating closest to their physiological maximum are benthic and mesopelagic diving sea lions—the Australian sea lion, the New Zealand sea lion (Costa *et al.*, 2004; Chilvers *et al.*, 2006; Fowler *et al.*, 2007; Villegas-Amtmann and Costa 2010) and the Northern fur seal when diving benthically. These sea lion species have the smallest populations, are classified as vulnerable or endangered (Tables 6 and 7) and exceed their cADL on between 45 and 57% of their dives (Fig. 4). They have previously been identified as operating near their physiological maximum (Costa *et al.*, 2004; Chilvers *et al.*, 2006; Fowler *et al.*, 2007). The tendency to exceed their cADL is likely to be a contributing factor to the slow recovery of populations of Australian and New Zealand sea lions, which all routinely exceed their cADL (Boyd and Croxall 1996; Chilvers *et al.*, 2006; Fowler *et al.*, 2007).

All other sea lion species demonstrate mixed strategies (Fig. 4). Sea lions are typically much larger than fur seals (Tables 6 and 7), and larger animals theoretically have the ability to make longer and potentially deeper dives (Villegas-Amtmann and Costa 2010; Weise *et al.*, 2010). However, we found that body mass was not correlated with the three behavioural parameters (TASD, dive depth and dive duration) nor the physiological parameters (DMR, cADL and TBOS). It is not surprising that neither TBOS or DMR were related to body mass, as we scaled values in our analyses, which also permits us to identify trends that were independent of body mass. For example, compared to other sea lion species, Galapagos sea lions have the highest mass-specific body oxygen stores [Villegas-Amtmann and Costa (2010), this study]. Galapagos sea lions are shown to have plasticity in their diving abilities linked to physiological (Villegas-Amtmann *et al.*, 2008) and environmental (Jeglinski *et al.*, 2015) differences. Individuals that specialize as benthic divers have higher TBOS than those classified as pelagic divers (Villegas-Amtmann and Costa 2010) and had different diet compositions as influenced by their foraging habitat (Jeglinski *et al.*, 2015). Similarly, for Southern sea lions, the longest and deepest diving animals had significantly larger TBOS than the shallowest and shortest duration divers (Hückstädt*et al.*, 2016) and the habitat they utilized also influenced their foraging behaviour (Baylis *et al.*, 2015b). Studies on the California sea lion demonstrate that there was not a significant cost of using a benthic diving strategy as the at-sea FMR did not differ for a deep diving or mixed strategy (McHuron*et al.*, 2016; McHuron *et al.*, 2018). Perhaps because of these physiological adaptations, these benthic diving sea lions were no more likely to exceed their cADL than pelagic divers. Though, future work should investigate the complex linkages between foraging strategy, environmental gradients and physiological constraints that influence the adoptation and change of foraging strategies (Jeglinski *et al.*, 2015).

The species least likely to be operating near their physiological maximum are the epipelagic diving fur seals. This includes the Antarctic, subantarctic, Galapagos, Guadalupe, Juan Fernandez and New Zealand fur seals. They are least likely to exceed their cADL (17–31% of dives, Fig. 3) and, with the exception of the Galapagos and Antarctic fur seal, have increasing or stable populations and are classified as least concern, despite all having been historically driven nearly to extinction by the fur trade (Table 6). Galapagos fur seal population dynamics are likely constrained by external forces, such as El Niño and fisheries, rather than physiological limitations related to foraging efficiency (Edgar *et al.*, 2010). Epipelagic fur seals dive to shallower depths for shorter durations and spend less TASD than benthic or mesopelagic foragers. However, it is unclear whether all epipelagic divers could or would adopt a benthic or mesopelagic foraging strategy. For example, the Galapagos fur seal is unlikely to exceed its cADL on a given dive (26%). Even in times of increased competition, in years of limited food availability and mass starvation, there was no evidence that these fur seals switched foraging strategies (Horning and Trillmich 1997). When Antarctic fur seals switched from primarily short and shallow pelagic dives to deep and long mesopelagic dives during periods of prey shortages, they increased the probabilty of exceeding their cADL on any given dive from 13.6 to 35.2% (Boyd *et al.*, 1994). The data available from the literature allowed us to test the impact of foraging strategy switching with one fur seal species—the Northern fur seal. There results demonstrate that the Norther fur seal exceeds its cADL when using a benthic diving strategy more often than not. Northern fur seal populations are currently in decline, seemingly in part related to changing prey distributions (Kuhn 2011). These environmental changes are likely to result in a change in Northern fur seal diving behaviour, pushing them to dive deeper and for longer (Kuhn *et al.*, 2010), and if that is not available to them, increasing their trip durations (Georges *et al.*, 2000a; Soto *et al.*, 2006).

**Table 6:** Phylogenies, morphometrics and demographics of females of nine species of fur seal and one sub species

Fur seals		Antarctic	Subantarctic	Northern	Galapagos	New Zealand	Guadalupe	Juan Fernandez	Cape	South American	Australian
Scientific name		*A. gazella*	*A. tropicalis*	*C. ursinus*	*A. galapagoensis*	*A. forsteri*	*A. townsendi*	*A. philippii*	*A. pusillus pusillus*	*A. australis*	*A. pusillus doriferus*
Mass	kg	22–50	25–67	30–50	27–33	~50	~50	~48	41–113	~40	41–113
Diet^*^		1, 4	1, 2, 3	1, 2	1, 2	1, 2, 5	1, 2	1	1, 2, 3	1, 2	1, 2, 3
Population	Size	~4 200 000	~400 000	~1 290 000	~15 000	~200 000	~18 000	~33 000	~2 000 000	~219 000	~120 000
	Trend	Decreasing	Stable	Decreasing	Decreasing	Increasing	Increasing	Increasing	Increasing	Increasing	Stable
	Status^#^	LC	LC	V	E	LC	LC	LC	LC	LC	LC
Environment		Sub-polar	Sub-polar	Sub-polar	Tropical	Temperate	Tropical	Tropical	Temperate	Temp/Trop	Temperate
References		1, 2, 3, 4	2, 5, 6, 7, 8	10, 11, 12	13, 14	15, 16, 17	18, 19	20, 21	10, 22, 8	23, 24, 25	26, 16, 27

^*^1. Fish; 2. Cephalopods; 3. Crustaceans; 4. Krill; 5. Birds; 6. Seals;

# LC, least concern; NT, near threatened; V, vulnerable; E, endangered

Mass is an estimate of a typical adult female. Diet is what is typically consumed and is not exhaustive. Population size has been derived from primary literature where available at the latest estimate and represents the total estimated population. Trend and status are from the IUCN red list. Environment is where the species is typically found latitudinally.

**Table 7:** Phylogenies, morphometrics and demographics of females of six species of sealion

Sea lions		Australian	New Zealand	Galapagos	Steller	Southern	California
Name		*N. cinerea*	*P. hookeri*	*Z. wollebaeki*	*E. jubatus*	*O. byronia*	*Z. californianus*
Mass		61–105	90–165	~77	~270	~144	63–95
Diet^*^		1, 2, 3	1, 2, 3, 6	1, 2, 3	1, 2	1, 2, 3, 6	1, 2
Population	Size	~13 000	~10 000	~10 000	~161 000	~445 000	~390 000
	Trend	Decreasing	Decreasing	Decreasing	Decreasing	Stable	Increasing
	Status	E	V	E	NT	LC	LC
Environment		Temperate	Temperate	Tropical	Increasing	Temperate	Temp/Trop
References		28, 29, 30	31, 32, 33, 34	13, 35, 47	36, 37, 38	39, 40, 41, 42	43, 44, 45, 46, 48

^*^1. Fish; 2. Cephalopods; 3. Crustaceans; 4. Krill; 5. Birds; 6. Seals;

# ^#^LC, least concern; NT, near threatened; V, vulnerable; E, endangered.

1. Guinet *et al.*, (1994); 2. Scientific Committee on Antarctic Research Expert Group on Seals (2008); 3. Campagna (2014); 4. Hofmeyr (2014); 5. Laws (1993); 6. Scientific Committee on Antarctic Research Expert Group on Seals (2004); 7. Bester *et al.*, (2006); 8. Hofmeyr (2015); 9. Hofmeyr *et al.*, (2006); 10. Gentry and Kooyman (1986); 11. Spraker and Lander (2010); 12. Gelatt *et al.*, (2015); 13. Alava and Salazar (2006); 14. Trillmich (2015a); 15. McKenzie *et al.*, (2007); 16. McIntosh *et al.*, (2014), 17. Chilvers and Goldsworthy (2015); 18. Gallo-Reynoso (1994); 19. Aurioles-Gamboa (2015b); 20. Francis *et al.*, (1998); 21. Aurioles-Gamboa (2015a); 22. Kirkman *et al.*, (2007); 23. Lima and Páez (1995); 24. Lima and Páez (1997); 25. Cárdenas-Alayza *et al.*, (2016); Kirkwood and Goldsworthy (2013); 27. Shaughnessy *et al.*, (2015)); 28. Lowther and Goldsworthy (2011); 29. Goldsworthy (2015); 30. Peters *et al.*, (2015); 31. Costa *et al.*, (2004); 32. Chilvers *et al.*, (2006); 33. Campbell *et al.*, (2006); 34. Chilvers (2015); 35. Trillmich (2015b); 36. Gelatt and Lowry (2012); 37. Maniscalco *et al.*, (2015); 38. Loughlin (2009); 39. Trillmich *et al.*, (1986); 40. Dans *et al.*, (2004); 41. Campagna (2014); 42. Thompson *et al.*, (2005); 43. Szteren *et al.*, (2006); 44. Carretta *et al.*, (2014); 45. Laake *et al.*, (2018); 46. Aurioles-Gamboa and Hernández-Camacho (2015); 47. Villegas-Amtmann *et al.*, (2017); 48. McHuron *et al.*, (2018).

Mass is an estimate of a typical adult female. Diet is what is typically consumed and is not exhaustive. Population size has been derived from primary literature where available at the latest estimate and represents the total estimated population. Trend and status are from the IUCN red list. Environment is where the species is typically found latitudinally.

Grouping otariids by species and foraging strategy does limit how far we can draw conclusions from our data. For each combination of species and foraging strategy, a single data point representing their dive and physiological parameters must be selected to be representative, even though these may have been measured several times. We are also limited by the behavioural and physiological variables that we have included. Other behavioural variables such as time spent at sea, or physiological variables such as body fat or thermoneutral zones, may be important for developing a deeper understanding of the differences in species or foraging strategies. In addition, we did not explore the impacts of environmental variables such as oceanography (Jeglinski *et al.*, 2015), climate change (Simmonds and Isaac 2007) or prey distributions (Horning and Trillmich 1999; Kuhn *et al.*, 2015) that are known to influence these parameters. Future work should seek to include these important variables.

An inability by some populations to display plasticity results in other responses to food shortages such as increasing the duration of foraging trips, (Boyd *et al.*, 1994) or spending more TASD (Georges *et al.*, 2000b), rather than increasing the depth or duration of dives. However, there is little evidence that for epipelagic foragers an increase in foraging effort can compensate for large-scale environmental changes. Despite overall increases in foraging effort, the growth rate of Antarctic fur seal pups reflects the food availablity of the year, where low food availability corresponds to poor growth and overall lower survival of pups (Trillmich *et al.*, 1991; Boyd *et al.*, 1994; McCafferty *et al.*, 1998). More widely, demonstrable environmental perturbations causing changes in fish abundance and latitudinal shifts in many of the ecosystems otariids inhabit are presenting them with new challenges, including prey scarcity and indirect resource competition (Bakun *et al.*, 2015; Sydeman *et al.*, 2015; Carroll *et al.*, 2016). The adaptive capacity of marine mammals depends, in part, on their ability to change their diet and or foraging behaviour in the face of these challenges. Prey becoming scarcer and more patchily distributed means that mothers may need to forage further from their rookeries or dive deeper and longer to obtain enough food to survive and provide for their pups (Boyd *et al.*, 1994). Foraging further from the rookery may have consequences on pup survival as the pups fast while mothers are at sea (Gentry and Kooyman 1986; Harcourt *et al.*, 2002), while increasing effort by diving deeper for longer is only an option if it is within the physiological capacity of the individual.

This study supports the theorizing of Arnould and Costa (2006) and Costa *et al.*, (2004) that some species may already be operating at their physiological maximum and therefore do not have the capacity to further adapt their foraging behaviour to a changing ecosystem. Here, the original conclusions by Arnould and Costa (2006) and Costa *et al.*, (2004) have been expanded, showing that switching from a pelagic to a benthic or mesopelagic foraging strategy significantly increases the likelihood of exceeding the cADL. Further, these results show that sea lions that switch strategies have physiological adaptations to do so. This study also provides further evidence that the Australian sea lion and New Zealand sea lion are also operating near their physiological limits. Where there is considerable variation in the experimental environment, variation among individuals combined with small sample sizes, it is useful to retest hypotheses to ensure that they stand up once additional studies add more species and increase the sample size. These are important findings to reevaluate with a rapidly changing climate that has increased the pressures these animals face, and the need for considered conservation measures are more urgent now than ever before. We caution that species thought to be able to change foraging strategy may not be able to do so due to the high cost of deep diving to undertake benthic and mesopelagic foraging. In the face of rapidly changing coastal ecosystems, female otariids face increasing constraints due to the central place foraging requirement arising from their income breeding strategy, that is, the need to return to feed their pup. For those species not able to push their operating constraints further, there may be untoward population consequences.

## Funding

This work was supported by a Macquarie University Research Excellence Support grant awarded to M. Ladds.
